# Na ion batteries: An India centric review

**DOI:** 10.1016/j.heliyon.2022.e10013

**Published:** 2022-07-20

**Authors:** Yogesh Singh, Rahul Parmar, Mamta, Sanju Rani, Manoj Kumar, Kamlesh Kumar Maurya, Vidya Nand Singh

**Affiliations:** a Academy of Scientific and Innovative Research (AcSIR), National Physical Laboratory, Dr. K.S. Krishnan Road, New Delhi, 110012, India; b Indian Reference Materials (BND) Division, National Physical Laboratory, Council of Scientific and Industrial Research (CSIR), Dr. K.S. Krishnan Road, New Delhi, 110012, India; c Elettra Sincrotrone, s.s. 14 km 163,500 in Area Science Park, 34149, Basovizza Trieste, Italy

**Keywords:** Sodium-ion batteries, India, Future of renewable energy, Cost analysis

## Abstract

Developing low-cost and safe energy storage devices is the primary goal of every country to make a carbon-neutral atmosphere by ∼2050. Batteries and supercapacitors are the backbones of future sustainable energy sources for electrical vehicles (EVs), smart electronic devices, electricity supply to off-grid regions, etc. Hence, these battery-dependent devices are substantially gaining the market. Although lithium-ion batteries account for powering most of these devices, lithium availability and price pose a severe problem since lithium resources are not abundant in nature. Thus, alternative research on sodium-ion or other multi-charged cations (Al^3+^/Mg^2+^/Ca^2+^/K^+^) based energy storage devices is needed to substitute lithium-ion batteries. India and many other countries have sodium in abundance. Sodium also has potential in designing and developing efficient charge storage devices. This review article discusses the status of sodium-ion battery research activities, cost, market analysis, and future strategies of the Indian government or private bodies, industries, and research institutes of India.

## 1 Introduction

Prof. Goodenough proposed the concept of lithium-ion rechargeable batteries in 1979 [1]. Subsequently, rechargeable lithium-ion batteries (LIBs) led to a boom in various industries pertaining to mobile telecommunication systems, such as laptops, digital cameras, smartphones, cordless electrical tools, electric vehicles, and other instruments. India indeed is one of the largest markets for these products and services. Due to its high energy density (ranging between 160 ‒ 220 Wh/kg), specific capacity, and long cycle life, LIBs have been more attractive than other batteries for a few decades. However, there are several concerns in the scientific community about LIBs, such as capacity fading, safety issues, and increased cost. Lithium-air (Li-Air), lithium-sulfur (Li–S), Na ion batteries (NIBs), and multi-charged cations (Al^3+^/Mg^2+^/Ca^2+^/K^+^) batteries, etc., are under development, which has the capabilities to replace the traditional LIBs [2]. Even the Li-Air and Li–S again require lithium metal as the main element, and lithium resources are very scarce. Thus, alternative cost-efficient NIBs would be the future energy storage device. The cost-effectiveness of sodium is well understood because sodium minerals are 20–30 times (Na_2_CO_3_, Na_2_SO_4_, and NaCl) cheaper than lithium [3]. Sodium resources are abundant in India from the sea sites, limestone mines in Rajasthan, Chhattisgarh, Jharkhand, etc. [4], making it a good choice for the Indian science community to work on NIBs. Yet, some practical factors must be considered to design efficient batteries beyond LIBs, such as NIBs with high redox potential, atomic mass, and greater ionic radius, thus affecting their overall specific capacity [5]. The cost of NIBs can be diminished by replacing the conventional copper current collector with an aluminum metal, which also makes NIBs lighter [6, 7].

Generally, hard carbon or nano-carbon-based composite materials are used in NIBs rather than traditional graphite as an anode [8]. For example, multi-layered graphene can host higher Na ions than graphite [9], which improves Na ion's storage capacity. Usually, the following materials (for NIB electrodes) have been used worldwide, (i) transition metal oxides-based cathodes, such as NaMO_2_ (M = Fe, Ni, Mn, Co, etc.) [10], (ii) polyatomic anion-based materials, i.e., NASICON-type Na_3_V_2_(PO_4_)_3_, Na_4_V_2_(PO_4_)_2_FO_2_ NaFePO_4_ and Na_2_FeP_2_O_7_ [11, 12, 13], (iii) transition metal fluorophosphate, and Prussian blue, i.e., Na_x_M [M′ (CN)_6_]_(1−y)._ zG type materials (here M and M′ are the transition metals, G is the neutral guest, i.e., H_2_O and y is the number of [M′ (CN)_6_]^n−^ vacancies) [14], (iv) organic compounds, i.e., azo (N=N), imine (C=N), carbonyl (C=O), thioketone/thioester (C=S), and free radical (N–O˙) reaction, etc. [15, 16, 17, 18, 19, 20].

(1) $$Na_{x}C_{6}+Na_{1-x}MO_{2}⇌NaMO_{2}+{\text{C}}_{6}$$

Additionally, the solid electrolytes make NIBs much safer for electric vehicles, geological surveys, defence equipment and provide an advantage of the molding in different shapes and sizes. Even though oxide and sulfide-based electrolytes are gaining more interest, high resistivity between electrode and solid electrolyte is the main hurdle that must be overcome [21]. Thus, NIBs are not just a theoretical concept; but an imminent success that can soon translate into a practical reality. This review article outlines the NIBs mechanism, components, cost analysis, and contribution made by the Indian scientific community specifically towards developing cathode and anodes. This review will thus be a torchbearer for the young researchers, as it sheds light on several aspects of NIBs and aids them in finding new collaborations. Basic cost comparison between LIBs and NIBs is also discussed.

## 2 Scenario of NIBs

**Figure 1:**
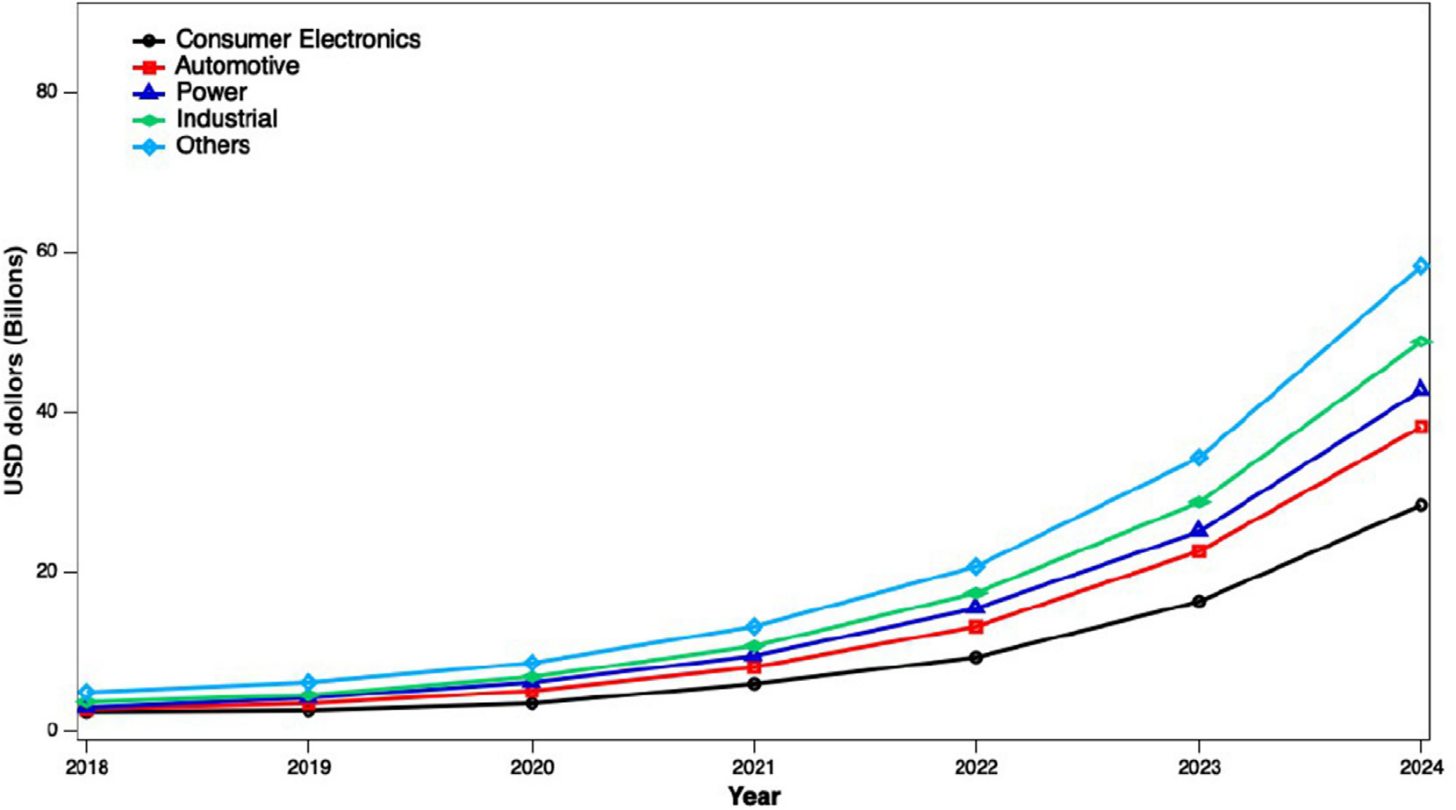
The overall demands (USD in billions) of Na ion battery MARKET by its application area year-wise (reprinted with permission from Ref. [25]).

A NIB uses sodium ions as charge carriers, which is relatively new, compared to other batteries. The salt used for the battery is much cheaper than any additional battery salt. The Ford motor company's first successful attempt for a sodium-based battery was undertaken in 1967, which used Na–S battery in their commercial vehicles [10, 22]. Because of the rapid success and practical applications of LIBs, NIBs were rejected. Recently, NIBs have emerged as an alternative to LIBs and have received significant attention from researchers worldwide. Recently, cathode materials prepared from organic material, transition metal oxides, and phosphates were introduced for NIBs. Simultaneously, the anode materials such as sulfides, organic compounds, and selective carbonic materials facilitated NIBs development. Apart from cathodes and anodes, some other materials are also required, such as additives, binders, and electrolytes that help design NIBs. Initially, handling sodium metal was difficult due to the less availability of efficient glove boxes and overall quality of electrolytes, making it challenging to study electrode performance. Composites of Sodium-lead as an anode and a P2-type Na_x_CoO_2_ were implemented as a cathode. Though it suffered from low discharge potential value of 3.0 V, it showed acceptable charge-discharge beyond 300 cycles, while its competitor carbon//LiCoO_2_ material exhibited a discharge voltage of around ∼3.7 V [23, 24]. Figure 1 shows the overall demand for NIBs in various fields from the last four years to the following three-year prediction worldwide.

## 3 Mechanism

Except for their ion carriers, the mechanism of NIBs and LIBs are almost similar. NIB also works on reversible chemical reactions, which occur at cathode and anode and provide electrical energy. The power density or specific capacity of any rechargeable battery depends on various factors, such as (i) available crystal structure volume (i.e., layered, spinel, monoclinic, orthorhombic, etc.) to host cations and (ii) stability of electrolytes (solid/liquid), cathode/anode materials at the applied potential value, elevated temperature, etc. Na^+^ ions travel through the cathode during the charging process at applied external potential, which provides a driving force for intercalation into the anode. Na ions are drawn out from the anode during the discharge process and re-intercalated into the cathode. The formula to calculate the specific capacity (in unit mAh/g) are shown in Eq. (2).

(2) $$Q=\frac{n.F}{3.6\;M}$$

**Figure 2:**
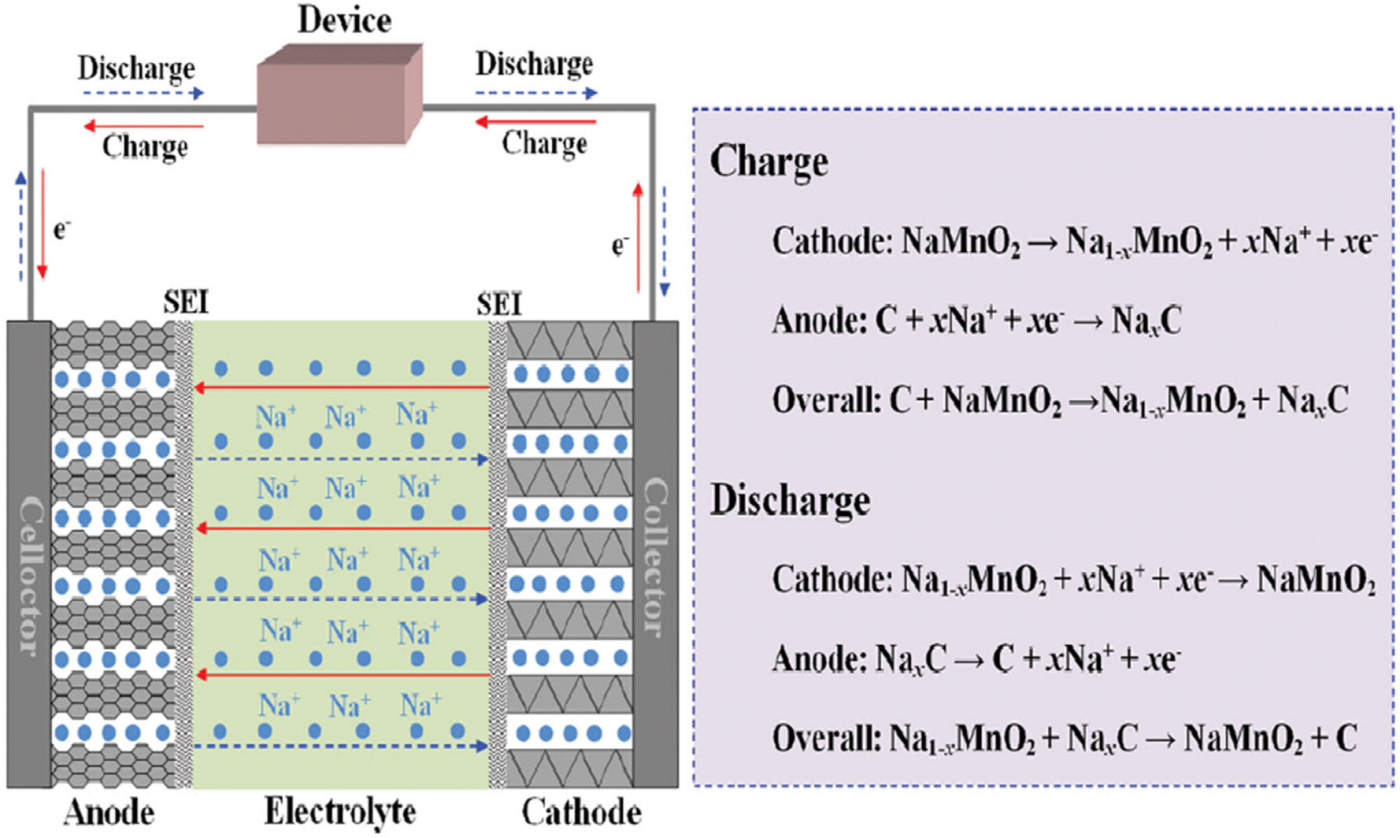
Schematic visualization of working principle and mechanism of Na-ion battery (NIB), reused with the permission [26].

Here, Q is termed as theoretical capacity, n is termed as the number of the transferred electron, M is termed as the molecular weight, and F is termed as the Faraday constant. A small M and high F means larger theoretical capacity. For example, NaFePO_4_ shows a higher theoretical capacity of ∼154 mAh/g than Na_2_FeP_2_O_7_ (97 mAh/g) because of its low M. The theoretical and experimental specific capacities of a designed battery have a considerable difference due to some irreversible chemical reactions at the surface of both electrodes, which is generally known as a solid permeable interface (SPI) at the cathode and solid electrolyte interface (SEI) at the anode. The role of SEI and SPI layers in NIBs performance is still under investigation for several electrode materials. Manganese oxides-based cathodes are widely used for LIBs and NIBs development after layered LiCoO_2_ cathode due to layered and three-dimensional structure phases, like alpha, beta, gamma or delta, etc. It is also a non-toxic and low-cost material. The experimental specific capacity for NaMnO_2_ was reported as ∼ 243 mAh/g, which can be improved by making composite with other transition oxides or nano carbons [6, 26, 27]. The manganese oxide cathode-based battery suffers from fast capacity fading due to well-known crystal structural instability (Mn^3+^ = Mn^2+^ + Mn^4+^) during the charge-discharge process and SPI layer formation on it, as it is widely studied for LIBs) [28, 29]. The charge-discharge mechanism of NIBs is shown in the left-hand side image of Figure 2, which includes the cathode (NaMnO_2_), anode (hard carbon), electrolyte, and current collectors. The individual chemical reactions at both electrodes and redox reactions in a NaMnO_2_-based cathode and carbon-based anode system are reported in the right section of Figure 2. Ronnie *et al.* reported an experimental study on the carbonaceous anode to test the impact of SEI dissolution and suggested that the primary reason for poor performance is the dissolution of active material and the formation of SEI [7, 30]. In many cases, the SEI layer contains the sodium hexafluorophosphate (NaPF_6_) immersed in a solution of alkyl carbonates as an electrolyte solution [5, 30]. Usually, the SEI layer has a higher thickness than the SPI layer due to different working potential values of anode and cathode and electrode-electrolyte interface.

## 4 NIB components

### 4.1 Carbon and its derivatives-based anode electrode

Generally, graphite is considered an active anode material for LIBs. Because sodium ion has a larger atomic radius than lithium, it cannot intercalate/de-intercalate easily between atomic layers of graphite. The hard carbon material is the best candidate for active anode electrodes for NIBs [31]. In 2003, NIBs containing hard carbon were reported, which provided ∼3.7 V discharge potential [32]. The solid form of carbon is the most suitable material for anode because of its good cycling stability and charge capacity retention. Several factors may affect NIBs performance and should be noticed, such as (i) crystal structural modifications after the intercalation/de-intercalation process of Na ions from anode material, (ii) chemical composition formation on the anode surface (SEI layer) after the charge/discharge process is responsible for capacity retention or fading of NIBs and (iii) crystal structural stability of anode materials at an operating temperature [33]. Several types of active anode materials generally used for NIBs are reported in Table 1.

**Table 1:** Active anode materials used for NIB along with reported specific capacity and maximum charge/discharge cycles.

Anode materials	Specific capacity (mAh/g)	Charge/discharge cycles	Reference
Red phosphorus/carbon composite	1890	30	[34]
Nitrogen-doped porous carbon nanosheets	349.7	260	[35]
Carbon nanosheets	255	210	[36]
Hollow carbon nanowires	251	400	[37]
Hollow carbon nanospheres	160	100	[38]

### 4.2 Transition metal oxide (TMOx) based cathode and anode materials

#### 4.2.1 Transition metal oxide (TMOx) based cathode materials

The cathode material is the main component that determines the voltage and capacity of NIBs. The host cation, i.e., Na ions, intercalate/de-intercalate into available active sites of the cathode crystal structure at a particular potential value. The selected cathode material should have some characteristic properties, such as (i) it should have enough space or volume into crystal symmetry to occupy the Na ions reversibly, (ii) cathode material should have high structural stability and minor deformation during the intercalation/de-intercalation process, (iii) chemically stable surface avoids the higher thickness of a solid permeable interface layer, which may block Na ion diffusion into the cathode, resulting in high-capacity retention. The selection of cathode material depends on its potential operating range, available crystal volume, life cycles, and operating temperature conditions. Several transition metal oxides (TMO_x_) based cathode materials are designed and selected for NIBs due to their unique properties like high stability, large working potential window, low cost, safe, available crystal volume, ease of synthesis, and abundance. Instead of metal oxides, the fluorinated transition metal or phosphates are better for efficient cathode materials for NIBs [39]. Most reported cathode materials are layered oxides such as Na*M*O_2_and Na*M*F, where *M* is Fe, Ni, Co, Mn, etc. Researchers have tried to improve the cathode performance via tailoring the polyanionic group. Some primary cathode materials are listed in Table 2. Layered Na_0_._62_ [Fe_0.5_Mn_0.5_]O_2_ shows promising electrochemical performance [40]. Calcium substitution also offers better results because of structural changes, as in the case of Na_5/8_Ca_1/24_CoO_2_ [41]. The different chemical compositions or stoichiometry of T*M*O_x_, fluorinated or phosphates-based cathode materials with their mixed oxidation states (during/after redox reaction), working potential window calculated theoretical and experimental specific capacity are reported in Table 2.

**Table 2:** Cathode materials with their calculated theoretical and experimental specific capacity within the potential working window for NIBs.

Cathode materials	Oxidation states	Voltage (V)	Theoretical capacity (mAh/g)	Experimental capacity (mAh/g)	Reference
Mn_x_V_2_O_5_/MWCNT	V^3+/4+/5+^	1.0–3.5	-	∼400	[27]
β-MnO_2_	Mn^3+/4+^	1.0–4.3	308	300	[42]
α-V_2_O_5_	V^4+/5+^	1.5–3.8	294	250	[43]
Na_0_._62_ [Fe_0.5_Mn_0.5_]O_2_	Fe^3+/4 +^ Mn^3+/4+^	1.5–4.3	263	185	[40]
Na_0.5_CoO_2_	Co^3+/4+^	2.7–3.5	263	140	[44]
Na_2/3_ [Co_2/3_Mn_1/3_]O_2_	Co^2+/3 +^ Mn^3+/4+^	1.5–4.3	258	180	[45]
Na_2/3_ [Ni_1/3_Mn_2/3_]O_2_	Ni^2+/4+^	2.0–4.5	258	162	[46]
Na_2/3_ [Ni_1/3−x_Mg_x_Mn_2/3_]O_2_	Ni^2+/4+^	2.0–4.5	258	145	[47]
Na_2/3_ [Ni_1/3−x_Al_x_Mn_2/3_]O_2_	Ni^2+/4+^	2.0–4.5	∼258	147	[48]
Na_2/3_ [Ni_1/3−x_Fe_x_Mn_2/3_]O_2_	Fe^3+/4+^Ni^2+/4+^	2.0–4.5	∼258	145	[48]
Na_0.7_ [Mn_0.65_Ni_0.15_Fe_0.2_]O_2_	Fe^3+/4+^Ni^2+/4+^	1.5–4.3	258	208	[49]
Na_2/3_ [Ni_1/3−x_Co_x_Mn_2/3_]O_2_	Ni^2+/4+^	2.0–4.5	∼258	144	[48]
Na_0.7_ [Fe_0_._5_Co_0.5_]O_2_	Fe^3+/4+^Co^3+/4+^	2.0–4.5	256	170	[50]
Na_5/8_Ca_1/24_CoO_2_	Co^3+/4+^	2.0–4.5	255	124	[41]
Na [Ni_0.25_Fe_0.5_Mn_0_._25_]O_2_	Ni^2+/4+^Fe^3+/4+^	2.0–4.5	240	140	[51]
Na [Ni_0.25_Fe_0.5_Co_0.25_]O_2_	Ni^2+/4+^Fe^3+/4+^Co^4+^	2.0–4.5	239	140	[43]
Na [Ni_0.5_Mn_0.5_]O_2_	Ni^2+/4+^	2.0–4.5	239	185	[52]
Na_1−x_NiO_2_	Ni^3+/4+^	2.0–4.5	235	145	[53]
NaFeF_3_	Fe^2+/3+^	1.5–4.0	198	128	[54]
NaMnF_3_	Mn^2+/3+^	1.5–4.0	198	<40	[55]
NaNiF_3_	Ni^2+/3+^	1.5–4.0	193	<40	[55]
Na_3_V_2_(PO_4_)_2_F	V^4+/5+^	3.0–4.2	156	87	[56]
Olivine NaFePO_4_	Fe^2+3+^	∼2.8	154	120	[57]
NaV_0.96_Cr_0.04_PO_4_F	V^3+/5+^	3.0–4.5	142	80	[58]
Na_2_FePO_4_F	Fe^2+/3+^	2.0–3.8	124	100	[59]

#### 4.2.2 Transition metal oxide (TMO_x_) based anode materials

The anode material for NIBs is not just limited to carbonaceous materials. Transition metal oxides like Fe_3_O_4_, Fe_2_O_3_, Co_3_O_4_, MnO, CuO, and NiO have shown promising results as anode materials for NIBs. However, the bottleneck for industrial applications is low reaction potential and low reversible capacity than the theoretical capacity. The first-ever reported non-carbonaceous transition metal oxide-based material was spinel-shaped NiCo_2_O_4,_ which showed a 200 mAh/g reversible capacity after initial discharge of 618 mAh/g [60]. Balaya et al. also used a Fe_3_O_4_ anode for sodium storage. The device showed a 643 mAh/g initial discharge capacity with an irreversible capacity of 50% and poor capacity retention [61]. α-MoO_3_ also showed promising results at a rate performance of 100 mAh/g with full capacity retention even after 500 cycles [62]. Few highly used anode materials are given below in Table 3.

**Table 3:** Anode materials with their experimental specific capacity and their capacity retention.

Material name	Specific capacity (mAh/g)	Cu Current density (mAh/g)	Capacity retention	References
NiCo_2_O_4_	200	NA	52% after 500 cycles	[60]
Fe_3_O_4_	643	NA	50% after 1100 cycles	[61]
α-MoO_3_	100	NA	100% after 500 cycles	[62]
Nano structured Co_3_O_4_	447	25	85% after 50 cycles	[63]
Ti-doped CoO	285	100	100% after 20 cycles	[64]
ZnO– Co_3_O_4_@CC	684	200	NA	[65]
CoO-Nano CNTs	450	NA	86.8% after 2000 cycles	[66]
Co_3_O_4_	500	89	NA	[67]
Porous CuO nanowires	303	50	47.3% after 50 cycles	[68]
Ni–NiO hollow nanoparticles inside porous carbon nanosheets (Ni–NiO/PCN)	235.4	1000	84.2% after 5000 cycles	[69]

### 4.3 Electrolyte, salts, and solvents for NIBs

The electrolyte is a medium that governs the power density by allowing ionic transport, most often Na ions transport in NIBs. The role of the electrolyte should not be ignored, as it is responsible for the lifespan and rate capability of the device, in addition to mechanical, electrochemical, thermal, and voltage stability. In NIBs, both aqueous and non-aqueous types of electrolytes are used. The most commonly used is the non-aqueous electrolyte of sodium hexafluorophosphate [70]. Electrolyte additives can favorably affect the performance of NIBs. The solvent is another essential component of an electrolyte, which carries Na ions and affects the diffusion rate. For the most suitable electrolyte, salts and solvents' properties can affect a cell's performance [43]. The different types of electrolyte salts and some common solvents with their melting and boiling temperature values used for NIBs are reported in Table 4. Where M_w_ is molecular weight, T_m_ is melting, and T_b_ is boiling temperature. While the ionic conductivity and electrochemical stability range of different types of electrolytes for NIB are shown in Table 5.

**Table 4:** The various electrolytes with their melting and boiling temperatures.

Salts	M_w_ (g/mol)	T_m_ (˚C)	References
NaClO_4_	122.4	468	[71]
NaPF_6_	167.9	300	[72]
NaBF_4_	109.8	384	[73]
Na_2_SO_4_	142.0	884	[74]
**Solvents**	**T**_**m**_**(˚C)**	**T**_**b**_**(˚C)**	**References**
PC	-48.8	282	[75]
EC	36.4	248	[74]
DEC	-74.3	126	[75]
DMC	4.6	91	[76]
DME	-58.0	84	[76]

**Table 5:** The ionic conductivity and electrochemical stability range of various types of electrolytes for NIB.

Electrolyte	Composition	Ionic conductivity σ @RT	Electrochemical Stability Range V vs. Na^+^/Na	References
Liquid (non-aqueous)	1 M Sodium perchlorate (NaClO_4_):Propylene Carbonate (PC)	6.4 mS/cm	0 V–5 V	[77]
	1 M Sodium perchlorate (NaClO_4_)-EC: PC	8 mS/cm	0 V–5.3 V	[77]
	1 M Sodium perchlorate (NaClO_4_)- EC: DME	12.55 mS/cm	0.5 V–4.5 V	[77]
	0.6 M Sodium hexafluorophosphate (NaPF_6_) - EC: DMC	6.8 mS/cm	1 V–4.5 V	[77]
Gel-polymers	PVDF-HFP:1 M sodium trifluoromethanesulfonate (NaCF_3_SO_3_) in EC: PC (1:1 vol%) + 3 wt% SiO_2_	4.1 mS/cm	N.A.	[88]
	EMTF:PVdF-HFP (4:1 w/w)+ 0.5 M NaTf	5.74 mS/cm	2 V–2.4 V	[89]
	PMMA-EC-PC-1 M Sodium perchlorate (NaClO_4_)	3.4 mS/cm	2 V–2.5 V	[90]
	PVdF-HFP- Sodium perchlorate (NaClO_4_)	0.6 mS/cm	0 V–4.6 V	[91]
Ionic Liquids	[Bmim][Br_3_]	8.93 mS/cm	NA	[85]
	BMP-TFSI + 1 M Sodium tetrafluoroborate (NaBF_4_)	1.9 mS/cm	NA	[92]
	(0.3)Na [FSA] [AS (4.5)][FSA]	1.3 mS/cm	NA	[93]

The different categories of electrolytes are explained in the following sections.

#### 4.3.1 Liquid electrolyte

Solid electrolytes are preferable for various reasons, including avoiding electrolyte leakage, low flammability, low electrode corrosivity, and the capacity to act as a separator between the electrodes. However, ionic conductivities of sodium ions into conducting solid electrolytes are typically lower than those of lithium ion-based conducting solid electrolytes due to bigger ionic radii. As a result, liquid electrolytes have recently been widely studied for NIBs due to the prospect of excellent ionic mobility in a liquid state. Ionic salt of sodium completely dissolves itself in an organic solvent. Based on the solvent utilized, these are classified into two types; aqueous electrolytes and non-aqueous electrolytes.

Non-aqueous electrolytes are produced by dissolving ionic sodium salts like NaClO_4_, NaPF_6,_ etc., in solvents like propylene carbonate (PC) and ethylene carbonate (EC). Ponrouch *et al.* discovered some electrolytes using three different ionic salts, NaPF_6_, NaClO_4_, NaPF_6_ and NaTFSI, dissolved in an organic solvent. More significant differences in the ionic conductivity and viscosity were found when 1M of NaClO_4_ was dissolved in a different solvent [76]. Bhide *et al.* presented electrochemical results of NaPF_6_, NaClO_4_ and NaCF_3_SO_3_ salt-based liquid electrolyte dissolved in a mix solvent of EC and DMC {where EC:DMC = 30:70 (wt.%)}. It was confirmed that NaPF_6_ in the solvent mentioned above shows satisfactory ionic mobility at various working temperatures, proving an ideal electrolyte for practical uses [77].

The main benefit of an aqueous electrolyte is its non-flammability upon being subjected to higher temperatures than other organic solvent-based liquid electrolytes. These are cheaper and ensure battery safety. In addition, they exhibit higher ionic conductivity than non-aqueous electrolytes [78]. Because of the electrochemical decomposition of water, electrode material selection is essential. J.F. Whitacre *et al.* reported large-format, low-cost energy storage systems following a set of Na interactive electrodes in an aqueous medium electrolyte with a neutral pH. λ-MnO_2_ and carbon were employed as an anode and a cathode. The neutral pH electrolyte reduced the corrosion at the interface of the electrode. The cell's performance was examined using 80 V electrodes, a 2.4 kW h battery pack, and a 1 M Na_2_SO_4_ electrolyte. Results showed excellent stability and cycle life [79]. In another work, a Na_2_NiFe(CN)_6_ cathode-based battery with NaTi_2_(PO_4_)_3_ as an anode and Na_2_SO_4_as an aqueous electrolyte showed an excellent cycle life having a theoretical energy density of 42.5 Wh/kg and 1.27 V output voltage. The 88% capacity retention of the initial value was recovered after 250 cycles [80].

#### 4.3.2 Solid electrolyte

**Figure 3:**
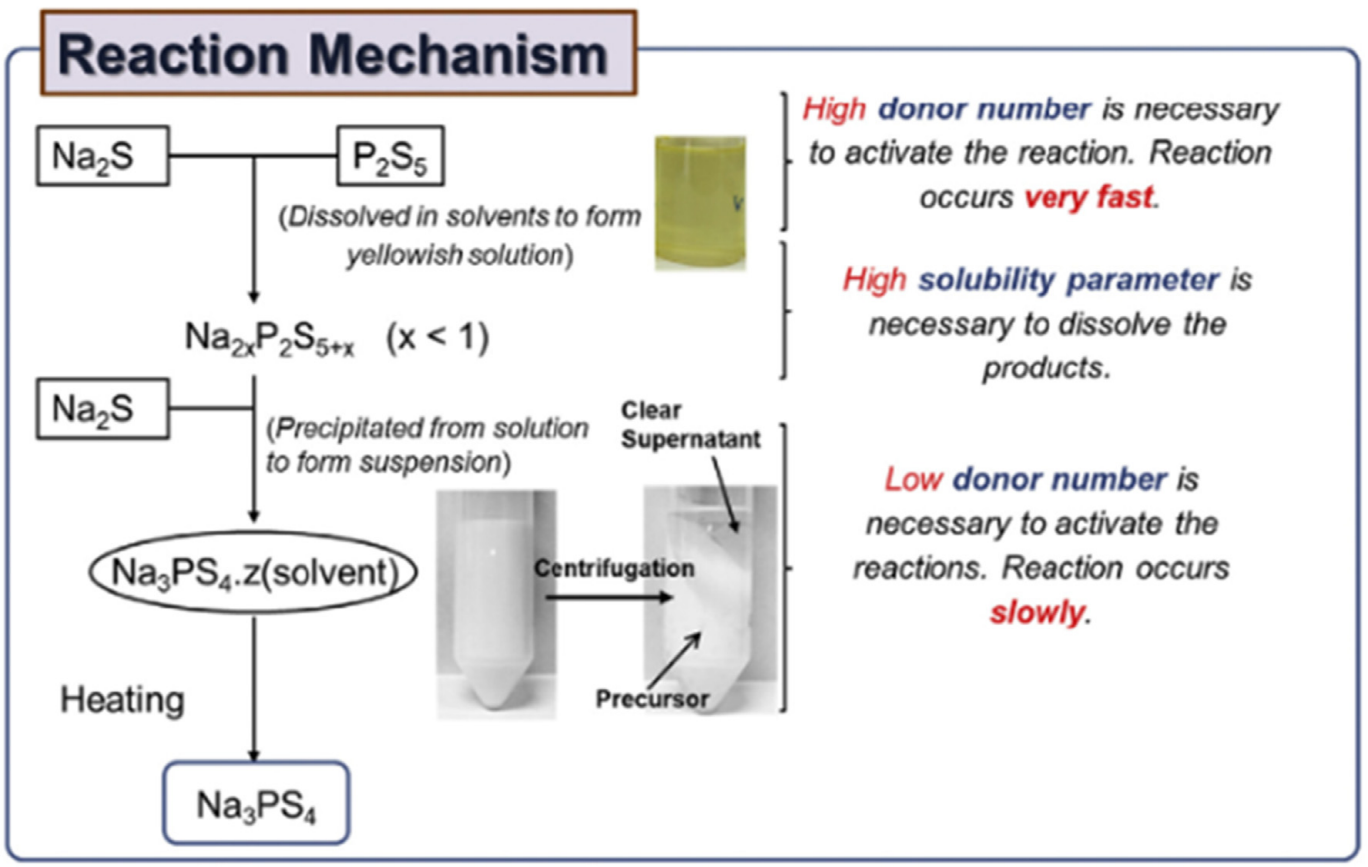
Schematic showing the reaction mechanism of c-Na_3_PS_4_ formation for Na–S battery (reprinted with permission from Ref. [84].

Solid electrolytes have attracted great attention among battery researchers as they may help avoid flammability in the battery. Mainly they are used in solid-state Na–S batteries, in which sulfur composite electrolytes are used. Solid sulfur composite works as active material in these types of cells. Hayashi and his group reported the formation of tetragonal Na_3_PS_4_ followed by heating Na_2_S–P_2_S_5_ and ball milling process at 270 °C and 420 °C temperatures, respectively [21]. Similar electrolytes, like Na_3_PS_4_–Na_4_SiS_4_, Na_3_P_1−x_As_x_S_4_ and Na_3_PSe_4−x_S_x_ were also reported in the literature [81, 82, 83]. Furthermore, after the precipitation, methyl acetate was employed as a reaction medium between Na_2_S and P_2_S_5_. The final product (c-Na_3_PS_4_) was collected, followed by centrifugation [84], as shown in Figure 3.

#### 4.3.3 Ionic liquid electrolytes

At room temperature, ionic liquids (ILs) or molten salts have minimal vapor pressure and deliver outstanding electrochemical stability. Protic and aprotic ILs are becoming attractive for NIB applications. Bagno *et al.* reported the ionic liquids based on imidazolium (3-methylimidazolium and 3-butyl-3-methylimidazolium) cation and trihalides as an anion. Compared to other typical imidazolium ionic liquids, such as chlorides, iodides, etc., trihalide-based ILs showed lesser viscosity, lower melting point, greater hydrophobicity, and conductivity of 8.9 mS/cm for butyl-3-methylimidazolium and 40 mS/cm for 3-methylimidazolium [85]. Monti *et al.* studied the imidazolium-TFSI and Sodium trifluoromethanesulfonimide (NaTFSI) for room temperature applications and reported anionic conductivity up to 5.5 mS/cm [86].

#### 4.3.4 Gel polymer electrolyte

**Figure 4:**
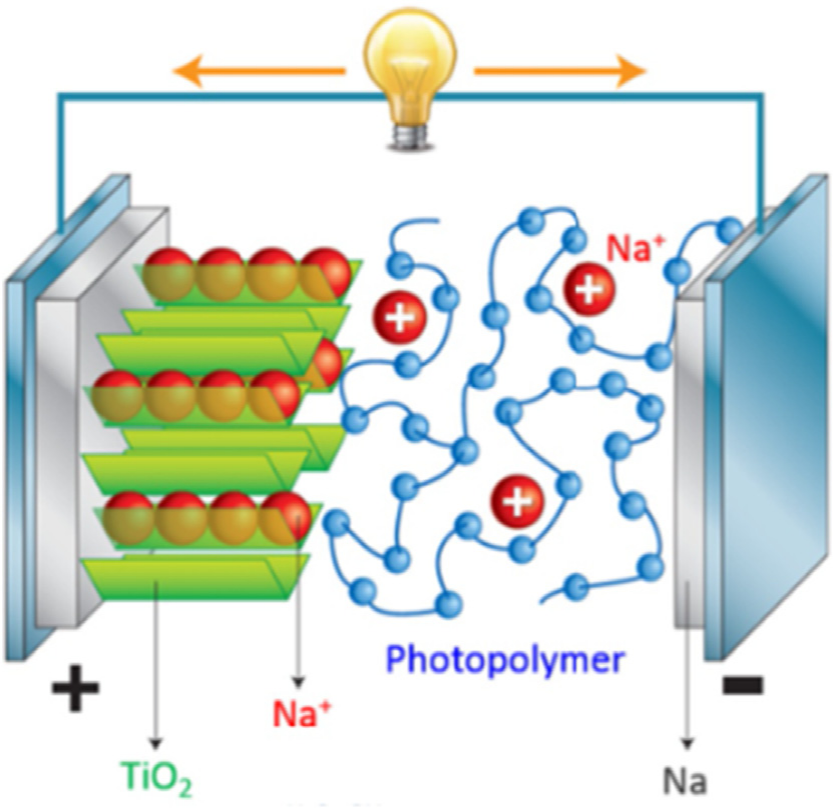
Schematic representation of NIB with a photopolymer electrolyte (reprinted with permission from Ref. [21]).

For large-scale grid support, battery durability and operating price are more important things to be taken into account than volumetric and gravimetric density. Recently, photo polymerized electrodes have been introduced for Na-ion batteries. The photopolymer electrolyte (shown in Figure 4) provides an overall light weight, solid-state construction, and low-cost fabrication with various sizes and shapes. Also, they are much safer for users, being non-corrosive, non-explosive, and having fewer internal circuits [21]. In a study, a three-dimensional polymer network is achieved, which is capable of Na ion transport and showed an electric potential range up to ∼4.8 V versus Na/Na^+^. Also, the high ionic mobility of 5.1 mS cm^−1^ was obtained. When the ionic mobility increased, GPE polymers showed high ionic conductivities. GPE may deteriorate more quickly due to a loss of liquid, which is usually reduced by high viscosity solvents. Yang *et al.* reported Na ion-conducting polymer-gel (PVDF-HFP) developed by simple phase separation method with 1M LiClO_4_ solution in EC/DMC/DEC by impedance method. These electrolytes behave dually like polymers and liquid electrolytes, increasing the strength and ionic conductivity. Time-dependent impedance tests were performed on a Na/PVdF-HFP/Na cell using Na metal-electrodes. Similar tests were performed on SS/PVdF-HFP/SS cells using stainless steel (SS) electrodes. The ionic conductivity of the gel polymer electrolyte in the temperature range from 25 °C to 75 °C was measured, and the ionic conductivity of 0.60 mS/cm was recorded at room temperature. Lonchakova *et al.* used the widely viable copolymer poly (acrylonitrile-co-methyl acrylate), propylene carbonate as a plasticizer, and electrolyte salts NaClO_4_ or NaPF_6_ were used to make an effective polyacrylonitrile-based gel-polymer electrolyte. The electrolytes showed ionic conductivities up to 1.8 × 10^−3^ S/cm and larger cation transference numbers up to 0.89. The conductivity follows the Arrhenius pattern with a 12–15 kJ mol^−1^ activation energy [87].

#### 4.3.5 Electroactive separator electrolyte

Electroactive separators electrolyte (ESE) has recently gained popularity for self-charging rechargeable batteries. Polytriphenylamine (PTPAn) and polyvinylidene fluoride (PVDF) are used as electroactive separator materials. The electroactive PVDF is a semi-crystalline piezoelectric polymer material with several sensor applications. It presents distinct α-, β-, and γ-type crystalline phases. β-is having the dipole moment of 8 × 10^−30^ cm. Several methods, including solution casting and spin coating, are used to produce the electroactive phases of PVDF. Janakiraman *et al.* characterized electrospun PVDF as an electroactive separator. The separator was submerged in a 1M NaClO_4_ DEC solution. Here, the electroactive separator Na ion cell consists of a cathode of Na_0.66_Fe_0.5_Mn_0.5_O_2_ and an anode of Na metal. Beyond ∼90 charge-discharge cycles, it showed a stable specific capacity with 92% of coulombic efficiency^.^ Results also showed an ionic conductivity of ∼7.38 × 10^−4^ S/cm [94].

## 5 Comparative cost analysis

### 5.1 LIB cost analysis

Vaalma *et al.* compared the cost of lithium and Na ion batteries using the BatPaC 3.0 model (Battery Performance and Cost analysis model) [6]. The BatPaC analysis was calculated for 110 g of lithium per kilowatt-hour (per European household requirement), considering the electrolyte and cathode. The mass of lithium salt equals 6,734 g of Li_2_CO_3_ (average USD 6.5 per kg in 2015), with a total lithium cost of $44. Another comparative report of LIBs with the lead-acid battery states that 40 W h (i.e., 40 W for 1 h) is generated by every one kg of lead-acid battery. LIBs can generate up to more than the earliest lead-acid battery [95]. The delivered specific power (the amount of energy a system contains compared to its mass, expressed in watt-hours per kg) is ∼180 W/kg, ∼245–430 W/kg, and ∼2–5 kW/kg for lead-acid batteries, LIBs, and NIBs, respectively. According to a scientific report, the costs of LIBs are ∼$150–300 per KWh, compared to the lead-acid battery, which costs $65 per kWh. With the expansion of the EV market by companies like BMW and Tesla, this price is expected to fall significantly. It has been predicted that the price could go below $100 per kWh in the next three years, and by 2030 it can touch ∼$50 per kWh [96]. As the lithium-ion market scales up, demand for lithium ores will increase. It can be easily understood that exceeding the lithium-based battery demand will push it to higher prices, and the technology will have to shift towards sodium for cheaper options.

### 5.2 NIB cost analysis

**Figure 5:**
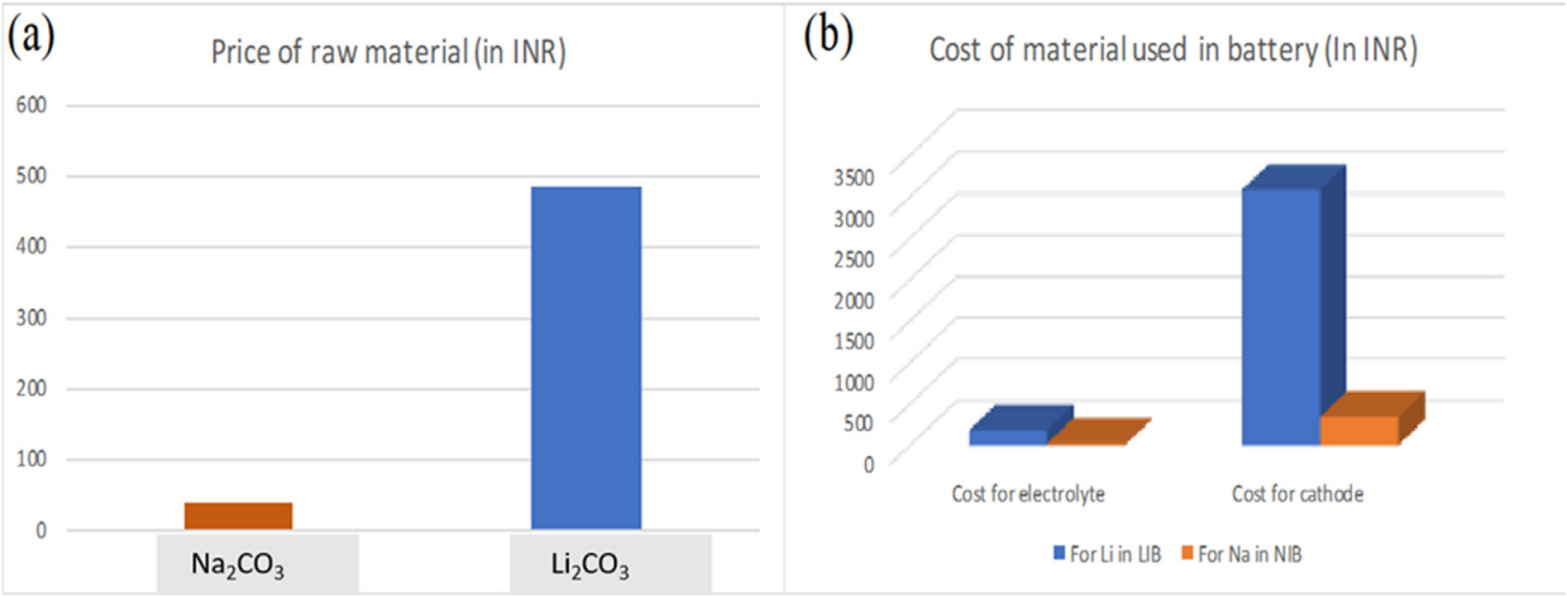
Raw material costs of Na_2_CO_3_ and Li_2_CO_3_ (a) and cost of electrolyte, and cathode materials, respectively (b). The cost analysis and price conversion have been done accoring to year 2020–21.

The cheaper sodium salt or raw materials make the NIBs less expensive than LIBs analogs. In the following text, we will discuss the cost comparison of Na_2_CO_3_ (price $ 0.50), considering the reference for sodium source in our calculations. Li_2_CO_3_ cathode material is more costly, costing $ 6.5 (as shown in Figure 5a). Vaamla and the team used a straightforward unique approach to compare the NIB cost with that of LIB. They took reference of LiMn_2_O_4_ (LMO) synthetic graphite anode-based battery and theoretically exchanged lithium with sodium. The theoretical calculation for NIB used aluminum as an anode. For 7 kW delivered power, 11.5 kW h LMO synthetic graphite battery, BatPaC recommends that 6 g and 104 g of lithium/kilowatt-hour are needed for the electrolyte and LMO cathode material, respectively. This total lithium will need 367 g of lithium carbonate, producing 69 g of lithium, costing 178.06 INR for an electrolyte. If we look for the cathode, it will take 6,367 g of Li_2_CO_3_ (1,196 g of lithium), which will cost $ 41.39. The team calculated that if the lithium is replaced with the same amount of sodium, it will cost $ 0.26 and $ 4.57 for electrolyte and cathode (shown in Figure 5b) (based on Na_2_CO_3_), respectively. It gives the manufacturer a $ 2.13 saving in the electrolyte and $ 36.82 in the cathode [6]. An overall decrease of almost ∼3.8% of the LMO in graphite cells and a ∼1.3% decrease in the final battery total cost [97, 98]. Going a step further, the team has also calculated if the replacement of lithium with sodium and copper with aluminum is considered altogether, it will achieve a 12.5% reduction in synthetic graphite cells and a 4.3% reduction in LMO graphite battery So, it can be very well seen that the NIBs can prove to be a cheaper substitute for LIBs in a country like India, with so many economic impediments [6].

## 6 Key areas

**Figure 6:**
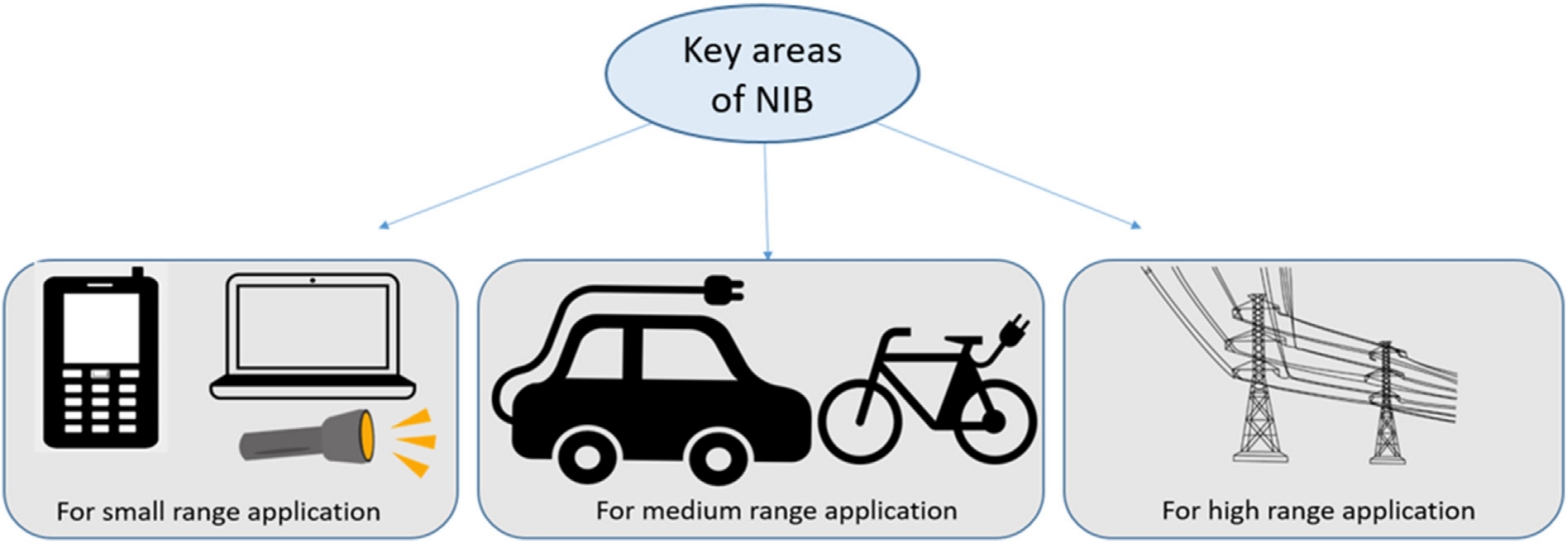
Critical areas for potential application of NIBs.

As a populous country with high energy demands, India requires a low-cost energy storage solution. NIBs are potential candidates to fulfill this requirement. Based on the different performance parameters, the usage of NIBs can be divided into three ranges (Figure 6) of applications, as explained below.

***Small range applications*:** portable electronic gadgets like laptops, torches, mobile phones, etc., require a battery capacity of 200–300 mAh/g, minimum cyclic life of ∼200 cycles or beyond, and capacity retention of more than 80%. With the pace of ongoing research, NIBs may soon replace LIBs and other battery types.

***Medium-range applications***: to operate an electric vehicle, high energy density and long cycle life (more than 1000 cycles) are necessary. Considering the recent results, these goals will not be so far to achieve.

**High range applications:** Grid-scale energy storage is required in various hydroelectric, wind, and solar power plants. It is the main requirement for long cycle life (>20,000 cycles) and storage efficiencies of >90%. New cathode material, like Na_3_V_2_(PO_4_)_3_ (NVP) and its composite, long cycle life (more than 30000), seems to be achievable [99].

## 7 Status of NIB in India

India has a vast energy storage market, second-largest after China in terms of energy consumption. Supposing that India wants to get away from its dependence on oil and wants its supremacy for cleaner and sustainable energy by moving towards renewable energy, it has to become reliant on imports as China owns 80% of the worldwide manufacturing of LIBs, relying on its vast lithium and cobalt availability [100]. China has 30 times more lithium reserves than the US, accounting for 46% of refined cobalt global production in 2016 [101]. Also, China has greater control over other battery-related materials like graphite. In such a scenario, if India wishes to show some LIBs manufacturing presence, it still has to depend on imports. If India has to succeed in its electric mobility strategy, it must rely on the batteries as much as possible. India now has a chance to get out of importing supply chain monopoly by adopting NIB technology without cobalt, copper, lithium, and graphite in sodium-based battery technology. The main advantage of Na ion technology is that it can be manufactured on existing infrastructure without additional capital investment. The only need is the material, which India has in surplus. To succeed, India needs to adopt a vertically integrated supply chain, i.e., the materials, battery capability, pack capability, and the manufacturers who can incorporate it entirely.

**Figure 7:**
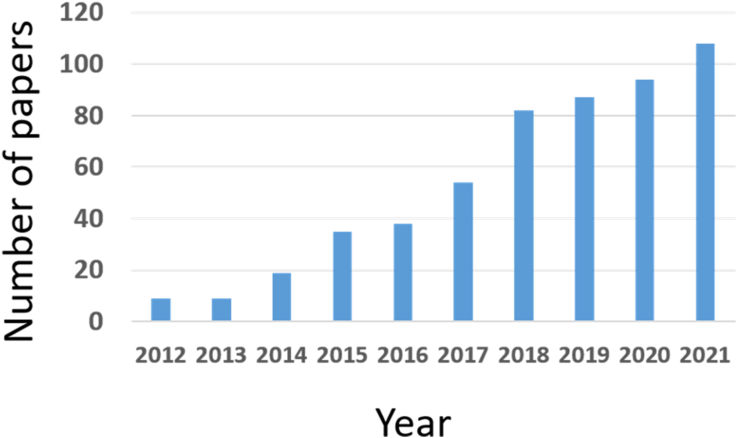
Number of the research articles published on the Na ion battery and year-wise in India (source-SCOPUS). Keywords used are “NIB,” “sodium-ion battery” “India,” and “Na ion battery.”

The fundamental phase of NIBs research in India started after the 20^th^ century, except for a few articles in the 90s. Major funding agencies in India are D.S.T (Department of Science and Technology), U.G.C. (University Grants Commission), and C.S.I.R (Council for Scientific and Industrial Research). While discussing individual scientific contributions, Barpanda from the Indian Institute of Science (IISc) Bangalore has the maximum contributions to his credit. Organization-wise, the Indian Institute of Technology (IIT) system leads from the front, followed by CSIR and IISc Bangalore. Figure 7 shows the number of research papers published year-wise by Indian researchers till August 2021.

NIBs are now emerging as a potentially viable option for Indian researchers for large-scale applications. Many research institutes, universities, and private firms are looking deeper into this. This research field has new dimensions after the “Make in India” initiative from the Indian government. India was late in the LIBs and other semiconductor-based device research fields, but it runs parallel to other countries' in research on NIBs. Recently, a UK-based company, “Faradion,” had done a partnership with an Indian company called Infraprime Logistics Technologies (IPL) to develop NIBs for commercial electric vehicles in India and set up an initial goal of manufacturing its initial target set at 1 GW h [102]. Concerned ministries have also come forward in this field. Centre for Materials for Electronics Technology (C-MET) under the Ministry of Electronics and Information Technology (MeitY) sought industrial partners to develop technology transfer to design and build machinery indigenously [103]. The giant project is achieving the goal of self-sustenance within five years. NIBs-based India research can be categorized into six major research activities in India (based on the significant work ongoing in different labs and universities), as shown in Table 6.

**Table 6:** Number of research problems the various Indian researchers is working on.

Sr. no.	Major research activity	Summary	Reference
1	Flexible electrode	Developed new flexible electrode materials for NIBs	[104, 105, 106, 107]
2.	Finding new electrolytes	The ion conduction mechanism of solid electrolytes plays a crucial role. Developing a new electrolyte for Na ion batteries can enhance ion conductivity.	[108, 109]
3.	New anode and cathode material from antimony-based materials or by their composites	Antimony-based material exhibits high capacity and cycling stability, a new plan with the sodium result in increased performance of Na ion batteries	[110, 111, 112, 113, 114]
4.	Finding new polyionic cathode material	The idea of finding polyanion cathode materials combining more than one kind of polyanion unit can show enhanced electrochemical properties.	[42, 70, 115, 116]
5.	Development of density functional theory (DFT) for NIBs designing	A theoretical model for density functional theory for Na ion batteries is also a hot topic in Indian research.	[116, 117]
6	Graphene-based NIBs	Graphene and its counterparts (holey graphene) are being used to demonstrate their applicability due to its electrical large surface area and high conductivity.	[105, 118, 119, 120, 121]

NIB technology will be well-maintained for EVs, requiring moderate energy densities, like rickshaws and scooters or electrical buses. The cost of NIBs will be approved, the same as lead-acid batteries, but provide three times the driving range. In keeping with the India FAME II initiative to assertively expand India's electric vehicle consumer base, NIBs will prove a better battery technology to catapult this vision to success.

## 8 Contributions from indian researchers

### 8.1 Early research (2012–2017)

The first research paper in India related to the Na ion battery was published in 2012 by M Sathiya. His team developed a new compound of layered Na, NaNi_1/3_Mn_1/3_Co_1/3_O_2_ (termed NaNMC), analogous to LiNi_1/3_Mn_1/3_Co_1/3_O_2_ (termed LiNMC). The material exhibited ∼120 mAh/g capacity analogous at 0.5 V Na in a limit of 3.75 V [122]. Later, B. Venkata Rami Reddy Jr. and Sukumaran Gopukumar reported a new kind of cathode material through the micro emulsion method, which exhibited the first cycle's ∼162 mAh/g discharge capacity when cycled between 2.0 - 4.2 V potential window, at a 0.1 C rate [123]. In 2014, Barpanda*et al.* developed a competent family of compounds suitable for Na ions insertion and synthesized pyrophosphate compound having formulae *Na*_*2-x*_*(Fe*_*1- y*_*Mn*_*y*_*)P*_*2*_*O*_*7*_*where 0 ≤ y ≤ 1* from the solid-state reaction method [124]. In the same year, Barpanda *et al.* extended the research on pyrophosphate cathodes and developed pyrophosphate oxyanion, t-Na_2_(VO)P_2_O_7_ along with Na_2_FeP_2_O_7_ and Na_2_MnP_2_O_7_ materials, which showed a reversible capacity (Q) of ∼80 mAh/g having a theoretical reversible capacity of 93.4 mAh/g, at 3.8 V Na/Na^+^ potential [125, 126]. Later, they investigated a sulfate family compound of Na_2_Fe(SO_4_)_2_·2H_2_O [127]. In the same duration, Suryavanshi *et al.* synthesized disordered graphitic carbon from pyrolysis of Indanthrone dye (ID). Such a half-cell assembly (Na/IDDGC) exhibited a ∼160 mAh/g reversible capacity at a current density of 25 mA/g [128]. In a search for anode material, Chaudhary *et al.* (2016) from IACS calculated the theoretical specific capacities for h-BN/black-Pn (Pn = Phosphorene , h-BN = Hexagonal Boron Nitride) anode material. The specific capacities of ∼607 mAh/g and 445 mAh/g for LIBs and NIBs were achieved in the theoretical calculation, more significant than existing commercial anode materials [129]. Singh *et al.* (2016) published a paper on polymer electrolytes, and their results indicated an excellent electrochemical potential window of 4.0–5.0 V and better cycling ability in the 2.7–1.6 V [130]. In 2016, Barpandadi made a comparison of the structural and electrochemical properties of two Fe-based sodium battery cathodes based on an oxide-based system and a polyionic-based system and described a strategy for improving energy density in both the systems [131]. Gosh *et al* (2016), worked on spinel-structured Li_4_Ti_5_O_12_ insertion anode material through an ultrasonic sonochemical route for Na and its lithium conterpart, where they found that the synthesized product with this material showed a reversible capacity of ∼45 mAh/g (at 0.9 V vs. Na^+^) [132]. Elizabeth *et al.* (2016) from CSIR NPL used prawn shells, later converted into carbon termed as PSC, for battery applications. The reported carbon (PSC) showed large amount of nitrogen content (5.3%) with macro, meso and micropores. PSC showed a ∼325 mAh/g specific capacity at 0.1 A/g and a ∼107 mAh/g rate capability of at 2 A/g for anodic application. The main focus of this study was to employ biowaste material for anodic application in Na ion battery [133]. Primarily developed cathode and anode material are summarized in Table 7 and Table 8 separately.

**Table 7:** Primary cathode materials developed by Indian researchers.

Material category	Material name	Potential range	Specific capacity (mAh/g)	Capacity retention	Structure type	Reference
Layered oxide	NaNi_1/3_Mn_1/3_Co_1/3_O_2_ or (NaNMC)	2.8 V	120 (0.1 C)	100% (50 cycles)	O3	[122]
Pyrophosphates	Na_2_MnP_2_O_7_	3.6 V	80	83% (15 cycles)	NA	[134]
pyrophosphate	Na_2_CoP_2_O_7_	3.0 V	80	100% (10 cycles)	NA	[135]
Layered oxide	Na_x_CoO_2_	2.7 V	162 (0.1 C)	80% (6 cycles)	P2	[136]
Pyrophosphate	Na_2_(Fe_1 − y_Mn_y_) P_2_O_7_	3.0 V	NA	NA	triclinic ​P-1	[137]
Pyrophosphate	Na_2_(VO)P_2_O_7_	3.8 V	80 (0.5 C)	NA	tetragonal	[125]
kröhnkite	Na_2_Fe(SO_4_)_2_·2H_2_O	3.25 V	70 (0.5 C)	NA	kröhnkite monoclinic structure	[127]
Mixed Metal Oxide	Na_0.67_Mn_0.65_Fe_0.20_Ni_0.15_O_2_	4.5 V	216 at C/15 rate	0.3% decay per cycle at C/15	P2-Type	[138]
Layered metal oxide	Na_0.6_Ni_0.25_Mn_0.5_Co_0.25_O_2_	3.6 V	125 at C/10	73.6% after 50 cycles	P3 type	[139]
	Zr–NH_4_V_4_O_10_	2.5 V	342 (at 0.1 A g^−1^)	66.6% after 500 cycles	1-D Nanobelts	[140]
Fluorophosphate	Na_2_FePO_4_F	2.0 V	85 ​(at a rate of 1 mA/cm^2^)	NA	NA	[141]
Laye|red oxides	SnO Coated Na_0.4_(Mn_0.33_Co_0.33_Ni_0.33_)O_2_	3.5 V	151 at 80 mA/g	80%. (100 cycles)	P2- type	[142]
Fluorophosphate	NaFe_1-x_(VO)_x_PO_4_	3.2 V	149.21	capacity retention (69.66%) even at 10C	Triclinic (P1) structure	[143]
Phosphate	(α-NaCoPO_4_) (also NCP)	2.5 V	36 at 0.1 C	capacity retention of 50% after 100 cycles	α- phase	[144]
Sodium chromium oxides	Na_0.95_CrO_2_	4.0 V	101	80%capacity retention after 50 cycles	O3	[145]
Sodium metal oxides	NaNi_0.5_Mn_0.3_Co_0.2_O_2_	3.6 V	∼136 at 0.1 C	63.9% at after 200 cycles0.1C rate	O3	[146]
Sodium metal oxides	Na_0.67_Mn_0.5_Fe_0.5_O_2_	2.5 V	∼166 at 0.1 C	68.9% after 100 cycles	P2-Type	[147]
Pyrophosphate	Na_2_FeP_2_O_7_	2.5 V	68 at 0.1 C	NA	O3	[148]

**Table 8:** Primary anode material developed by Indian researchers.

Material	Potential (V)	Specific capacity (mAh/g)	Capacity retention (%)	Structure type	Reference
IDDGC (Indanthrone derived disordered graphitic carbon)	2.5 V	160 at 25 mA/g	67% after 500 cycles	Layered	[128]
Li_4_Ti_5_O_12_	2.5 V	45	80% of initial capacity 50 cycles (at C/20)	cubic spinel structure	[132]
N-doped hierarchically porous carbon	3.0 V	325 at 0.1 A/g	NA	porous structure	[133]
Carbon Nanoparticles (CNPs) from ​coconut oil	3.0 V	277 at100 mA/g.	78% after 20 cycles	N.A.	[149]
nutshell-derived carbon (NDC)	3.0 V	257 at 50 mA/g	97% retention at 2 A/g	Hard Carbon	[150]
TiS_2_	2.6 V	∼146 at 0.1 C rate	NA	pure phase single crystals	[151]
Na_2_Ti_6_O_13_	2.5 V	40 mAh/g at 0.83 V (vs. Na/Na+)	NA	monoclinic structure with *C*2/*m* symmetry	[152]
Na_3_V_2_(PO_4_)_3_ molecule wrapped by carbon made from human hair NVP/HHC	2.5 V	158 mAh/g, (50 mA/g current density)	95% (after 100 cycles, current density of 2 A/g)	graphene sheet-like structure	[153]
N-doped spherical carbon particles	2.5 V	238 mAh/g after 500 cycles at 0.5 A/g	69.3% (250 cycles at 2.0 A/g)	conjugated honeycomb-like structure	[154]
Dual core-shell Fe_3_O_4_(PPy) composite	2.5 V	68 mAh/g at 0.1 A/g, after 60 cycles	NA	Spherical nanoparticles	[155]
N-doped carbon-nanosheets	3.0 V	150 mAh/g at 50 mA/g	80% After a specific current of 250 mA/g, 350 cycles	graphene oxide-like structures	[156]
WS_2_ ​nanosheets	3.0 V	400 mAh/g, at 1C rate	NA	hexagonal and trigonal nanosheets	[157]
Sb_2_O_4_/C	2.5 V	935 ​mAh/g At a current density of 0.1 ​A/g	97.8 % of the initial charge capacity after 125 cycles	Nanorod array	[158]
Boron doped graphene quantum dot (GQD)	2.5 V	310 mAh/g at a specific current of 50 mA/g	95.7 % After 500 cycles	zero-dimensional carbon nanostructures	[159]
zirconium doped hydrogenated Na_2_Ti_3_O_7_ (HNTOZr)	2.75 V	∼200 mAh/g at a current rate of 200 mA/g	85% capacity retention after 2500 cycles with more than	layer structured nanorods	[160]
Sb_2_Se_3_-rGO	2.5 V	550 ​mAh/g at a specific current of 100 mA/g	100 % capacity retention after high current cycling involving a 2 ​A/g	1D-Nanostructures	[161]
GCNT/S (sulfur, graphene CNT composite)	2.5 V	510 mAh/g, at 50 mA/g current density	0.037 % decay per cycle (till 600 cycles)	Nanotubes	[162]
SnO_2_	2.0 V	488 mAh/g (at 20 mA/g)	96% After 200 cycles	tetragonal rutile structure	[163]
BFHC-NC_5_	2.5 V	discharge/charge capacities of 413/358 ​mAh/g	86.6 % for the first cycle. For 500 cycles	micro-nano structured pores	[164]
Sb_2_O	2.5 V	623 mAh/g, 50 mA/g ​current density	65 %, after 200 cycles	cubic structure	[165]

Janakiraman *et al.* worked on electrospun polyvinylidene fluoride (PVDF) applications in electroactive separators, and their XRD results confirmed the material has a high porosity phase with an intrinsic β-phase. Under ambient conditions, the material showed ionic conductivity of ∼7.38 × 10^−4^ S/cm. When Na_0·66_Fe_0.5_Mn_0·5_O_2_ was used as a cathode and the separator was used, it achieved 92% coloumbic efficiency [166]. Gaddam *et al.* reported the carbon nanoparticles (prepared from biomass) for anode application in Na ion battery. They used carbon nanoparticles (CNPs) and surface-carboxylated nanoparticles (modified with piranha solution called c-CNPs). CNPs and c-CNPs showed discharge capacities of ∼277 and ∼278 mAh/g in the second cycle at a current density of ∼100 mA/g, ∼217, and ∼206 mAh/g, respectively, over the 20^th^ cycle [149]. In 2017, M. Wahid *et al.* from CSIR-NCL used hard carbon derived from the walnut shell for efficient Na ion intercalation/deintercalation. The Walnut shell was cleaned using acid and underwent high-temperature pyrolysis at 1000 °C, and milled. Such pyrolyzed hard carbon gave a larger inter-planer spacing than that of graphite. The hard carbon was found convenient for the intercalation/deintercalation of Na-ions. The above-mentioned derived carbon showed a reversible capacity of ∼257 mAh/g at a current density of ∼50 mA/g [150]. In 2018, Apoorva *et al.* synthesized TiS_2_ ​using the chemical vapor transport deposition method to insert it into Na-ion and lithium-ion. The Na/TiS_2_ ​cell displayed a capacity of ∼146 mAh/g ​at a 0.1 C rate corresponding to ∼0.61 mol [151]. In the same year, G Venkatesh *et al.* prepared Na_0.67_Mn_0.65_Fe_0.20_Ni_0.15_O_2_ from FeCO_3_ microspheres by thermal decomposition to convert it into oxide then oxide was thermally fused with Na_2_CO_3_. Such resulting oxide material showed an initial discharge capacity of ∼216 mAh/g. The decay rate of ∼0.3% on each cycle at the C/15 rate was observed but increased to 0.9% after 100 cycles [138].

In 2017, Dwivedi *et al.* brought a conference paper on alluaudites Na_2_M_2_(SO_4_)_3_ (where M = Co,Fe,Mn,Ni) class of material as sodium ion insertion material [167]. They reported on Na_2+2x_Co_2−x_ (SO_4_)_3_ (x = 0.16) material for cathode application with monoclinic structure and C_2/c_ symmetry. They further did the DFT investigation of the same material [168]. In the same year, Swatilekha Ghosh *et al.* reported on nanostructured Na_2_Ti_6_O_13_ as anode material for Na-ion batteries. The compound exhibited a theoretical capacity (ca. 40 mAh/g) involving a Ti^3+^/Ti^4+^ redox potential centered at 0.83 V (vs. Na/Na^+^) with excellent reversibility [152]. S. Karuppiah, S. Vellingiri, and K. Nallathamby (2017) from CSIR-Central Electrochemical Research Institute used carbon derived from human hair (named HHC or human hair-derived carbon) and Na_3_V_2_(PO_4_)_3_ composites anode material for Na ion battery. Rate capability was 2 A/g up to 500 cycles, which was better than the previously reported values [153]. S. Maddukuri, P. Valerie, and V. V Upadhyayula (2017) prepared Na_0.6_Ni_0.25_Mn_0.5_Co_0.25_O_2_ using a novel co-precipitation method. Electrochemical results showed that when the material used as a cathode it exhibited a reversible discharge capacity of 105 and 130 mAh/g at a C/10 rate. A coloumbic efficiency of nearly 99 % was observed for the layered material [139].

In 2017, A. Sarkar, S. Sarkar, and S. Mitra worked on doped ammonium vanadium oxide for Na ion battery application. They tested it against sodium titanium oxide (NTO) anode for full cell performance. The material showed a discharge capacity of 342 mAh/g at a 0.1 A/g current rate. The complete cell retained 94% capacity after 400 complete cycles and showed its possible application in a low-powered LED table lamp [140]. In the same year, V. Selvamani *et al.* made nitrogen-rich porous spherical carbon particle (interlayer distance 0.377 nm) with a large surface area (390 m^2^/g) using a simple pyrolysis method. For NIB application, the material exhibited a stable reversible capacity of about 238 mAh/g for the studied 500 cycles at 0.5 A/g. The steady-state cycling performance was 165 mAh/g even after 250 cycles at 2.0 A/g [154]. Harish Banda *et al.* tried to work on the low reduction potential issue. They tried to tune the redox properties of perylene diimides (PDIs) as a cathode material of sodium-ion batteries (NIBs). Doping some electron-withdrawing groups in perylene diimides tuned the discharge potential from 2.1 to 2.6 V versus Na^+^/Na (sodium intake of ∼1.6 ions/molecule) [169]. In November 2018, P Barpanda and his colleagues experimented to discover a cathode for sodium ion-based batteries. They discovered that for getting a reversible capacity of more than 85 mAh/g (at a rate of 1 mA/cm^2^), solution combustion synthesized Na_2_FePO_4_F was a very effective cathode. When NASICON-type NaTi_2_(PO_4_)_3_ is used as an anode with Na_2_FePO_4_F in a full aqueous cell, it gave a reversible capacity of 90 mAh/g [133]. In 2019, G. S. Shinde *et al.* from P. Barpanda's group prepared a new layered sodium iron phosphate phase [Na_3_Fe_3_(PO_4_)_4_] compound using combustion. Rietveld's analyses showed phase purity and generation of the monoclinic framework with C2/c symmetry [170]. In 2020, Vineet Shukla *et al.* from IIT Kharagpur manufactured a dual core-shell Fe_3_O_4_@C@polypyrrole (PPy) composite using a two-step method. They got outstanding electrical conductivity between Fe_3_O_4_ nanoparticles and PPy polymer from the carbon layer during their work by choosing a suitable ratio of Fe_3_O_4_/C and PPy. They used spectroscopic and microscopic techniques to specify this dual-core during the experiment. Moving forward, they used this composite in Na ion battery applications. They explored that at a voltage range of 0.01–2.5 V, this composite gave 64 mAh/g discharge capacity (100 cycles at a current density of 0.1 A g^−1^). At the current density of 1 A/g, its discharge capacity becomes 68 mAh/g (at 60 cycles) [155].

### 8.2 Progress during 2018–2020

In Feb 2020, Pankaj Srivastava and Sneha Upadhyay from IIITM Gwalior experimented with designing and discovering a two-dimensional layer of antimony (Sb) used as an anode. They calculated the adsorption energy charge transfer and density of states between Sodium (Na) atom and antimony. According to their study, after adsorption, antimony showed metallic behavior suitable for Na ion batteries. Antimony has high effective charge transfer and metallicity, which gives it better conductivity. Throughout sodiation, it is supposed that the antimony electrode would be stable. Their research forecasted that antimony has a higher specific capacity of 421.63 mAh/g. Sodium has a 0.12 eV lower activation energy barrier than the graphite anode and other materials. In short, their research concluded that antimony could be used as a very effective anode due to its high specific capacity, less expansion, and low diffusion barrier [171]. A. K. Radhakrishnan, S. Nair, and D. Santhanagopalan from the Centre for Nanosciences and Molecular Medicine worked on N-doped carbon nanosheets. They used it as an anode (in lithium-ion and Na ion batteries). They got a reversible capacity of approximately 500 mAh/g and 250 mAh/g at a specific current of 100 mAh/g and 500 mAh/g, respectively, after 600 cycles. Both lithium-ion and Na ion cells in nanosheet gave a low voltage profile, and it was as soft as carbon, so it is safe from metal plating and dendrite formation [156]. Joshua *et al.* from Chikkaiah Naicker College, Erode, Tamilnadu, worked on a P2-type Na_0.4_(Mn_0.33_Co_0.33_Ni_0.33_)O_2_. They manufactured it using the hydrothermal method and used it as a cathode in NIBs. Their research improved the cycling stability of NIBs by placing a layer of SnO on the surface of Na_0.4_(Mn_0.33_Co_0.33_Ni_0.33_)O_2._ Moreover, they also came to know by XRD and SEM analysis that the layering of SnO has not affected the morphology of Na_0.4_(Mn_0.33_Co_0.33_Ni_0.33_)O_2_. According to their research, at the voltage range of 0–3.5 V, this material gave a reversible capacity of 141 mAh/g, and after 100 cycles, it showed an adequate capacity of up to 80%. The movement of Na ions is sped up and electrolyte decomposition declined due to the layering of SnO on the surface of P2-type Na_0.4_(Mn_0.33_Co_0.33_Ni_0.33_)O_2_. This was the reason for the excellent electrochemical performance of NIBs [142]. In 2020, S. Gandi, V. K. Katta, D. P. Dutta, and B. R. Ravuri from GITAM University, Hyderabad, experimented on a mixed polyanion NaFe1−x (VO)_x_PO_4_ material and got an adequate specific capacity and capacity retention in Na ion batteries. Their experiment used NaFe_1−__x_(VO)xPO_4_ type ceramic cathodes in NIBs. This material NaFe_0.5_(VO)_0.5_PO_4_ (x = 0.5 mol%) gave the maximum capacity for the 1^st^ and 20^th^ cycles at 3.2 V and 2.8 V, respectively. Moreover, at 10 C, NaFe_0.5_(VO)_0.5_PO_4_ (x = 0.5 mol%) showed 69.66% capacity retention, and hence their work proved that for large-scale economic NIB mixed polyanion NaFe_1−__x_ (VO)_x_PO_4_ ceramic is an effective cathode [143]. In 2020, Gupta *et al.* worked on computational studies of PEO_3_-NaClO_4_-based solid polymer electrolytes for Na-ion batteries. Their work explained the changes in putting Na salt into polyethylene oxide PEO polymer. For computation, they took three monomer units of PEO and one molecule of NaClO_4._ During their work, Hirshfeld population analyses have been used to study the atomic charge distribution of each atom density of state (DOS) and partial density of state (PDOS), which helped in electronic observations. The DOS study observed the forbidden energy gap (PEO)_3_ with and without NaClO_4_. This energy gap declined by 1.5 eV for polymer alone. The results confirmed the increased sodium cation and ionic conductivity [172]. In 2020, P Sharma *et al.* from IIT Kharagpur worked on a new method to prepare tungsten disulfide (WS_2_). They used the wet synthesis method to produce (WS_2_) in an enormous amount. Moreover, their work also revealed that sulfurization of WO_4_^2-^ occurs through several stable intermediates such as WOS_3_^2-^ and WO_2_S_2_^2-^. To reduce the pH, sulfurization is obtained through H_2_S. Watching the complete process continuously was crucial as it was vital for product purity and yield moving forward. Using this material in LIBs and NIBs batteries as an anode, they obtained a specific capacity of 400 mAh/g at 1 C. Even at a 5 C rate (2.1 A g^-1^), the capability of pristine, bulk, up-scalable WS_2_ was very high (250 mAh/g) in LIBs [157].

In 2020, Arjunan *et al.* from Alagappa University constructed a polymer poly (vinylidene fluoride) membrane–silicon dioxide (PVdF-SiO_2_) using a simple phase inversion technique. Due to its higher dielectric constant value of 8.4, PVdF is used as the high-porous polymer electrolyte membrane. The membrane was characterized for morphology, porosity, and electrochemical properties. The capability of the separator membrane was examined with the help of an electrolyte solution of 1M NaPF_6_. Furthermore, a temperature-dependent ionic conduction test was carried out. This membrane showed 4.7 × 10^−2^ Scm^−1^ maximum ionic conductivity at room temperature. The membrane was used with sodium P2-type cathode material, which gave 178 mAh/g of initial discharge capacity at 0.1 C between 2 and 4 V. Moving forward using PVDF-SiO_2_ composite separator membrane, it showed Columbia efficiency and capacity retention after 50 cycles of 98% and 81%, respectively [144].

In September 2019, Dubey *et al.* from NIT Kurukshetra, using a conventional solid-state reaction technique, designed solid-state electrolytes Na_2_MTeO_6_ (M = MgNi and MgZn). XPS and XRD showed the pure hexagonal layered P2-type structure of this material. Raman and FTIR spectroscopy was used to study bending and stretching modes for Te–O and other oxides of different metals. This electrolyte showed ion transport properties. AC impedance spectroscopy with the classical brick layer model was used for its electrical properties. During experimentation at 20 °C, Na_2_MgNiTeO_6_ showed specific gain conductivity of 2.13 × 10^−5^ Scm^−1,^ and Na_2_MgZnTeO_6_ gave specific grain conductivity (σ_g_) of 0.90 × 10^−5^ Scm^−1^. Moreover, below 30 °C, both Na_2_MgNiTeO_6_ and Na_2_MgZnTeO_6_ showed activation energies of 0.59 eV and 0.36 eV, respectively. Thus, it was shown that this electrolyte could be used in Na ion batteries for its good performance [173].

In 2020, Subadevi *et al.* from Alagappa University researched the effect of downsizing the maricite α-phased sodium cobalt phosphate (α-NaCoPO_4_) in a Na ion battery. They used α-NaCoPO_4_ in Na ion batteries and revealed that this material showed redox activity at 2.33 and 4.3 V. Moreover, NCP/C showed reversible intercalation and a discharge capacity of 36 mAh/g at 0.1 C, and after 100 cycles in sodium half cells, its capacity retention became 50%. To make these particles nanosized, α-NaCoPO_4_ was ball-milled with NCP/C. The material showed an orthorhombic crystal structure, which was verified using powder X-ray diffraction. Furthermore, X-ray photoelectron spectroscopy (XPS) was used to prove the purity of the material. They used SEM and TEM studies to analyze the morphology and particle size of the materials [174].

In 2020, H. Verma, K. Mishra, and D. K. Rai from Jaypee University researched a membrane sodium ion-conducting nanocomposite polymer electrolyte. This membrane consisted of TiO_2_, which is a dispersed membrane of poly (vinylidenedifluoride-co-hexafluoropropylene) (PVdFHFP) soaked in a liquid electrolyte of sodium hexafluorophosphate (NaPF_6_) in ethylene carbonate (EC) and propylene carbonate (PC). The phase inversion method was used to disperse TiO_2_. In addition, X-ray diffraction, Fourier transforms infrared spectroscopy, and scanning electron microscopy was used to study this membrane's structural and morphological characteristics. The membrane was highly porous and had maximum porosity of 72% and 270% electrolyte uptake during experimentation.

Moreover, they observed ionic conductivity of this membrane in which TiO_2_ has various concentrations in complex impedance spectroscopy. They also obtained high ionic conductivity of about ∼1.3 × 10^−3^ Scm^−1^ at room temperature. Ionic conductivity also showed VTF behavior. A complex impedance cyclic voltammetry and dc polarization were used to study ion transport numbers. The membrane showed predominantly ionically conducting behavior and a Na ion transport number of about ∼0.31. The cyclic voltammetry showed it was electrochemical stable at 3.5V [175].

In 2020, Dutta from Bhabha Atomic Research Centre, Mumbai, worked on a material known as Sb_2_O_4_ and biomass-derived mesoporous disordered carbon. It was used as an anode in Na ionbatteries. Sb_2_O_4_ had an excellent theoretical capacity of 1227 mAh/g as an anode in NIB. On the other hand, its electrical conductivity and reversibility were not good, so she researched to overcome these limitations. The Indian blackberry seeds biowaste was used to prepare disordered carbon at a meager cost. X-ray diffraction (XRD), Raman spectroscopy, and electron microscopy techniques were used for classifying disordered carbon composite. The Brunauer-Emmett-Taylor (BET) method was used to study the porosity of the materials because it plays a crucial role in sodium ion transport. Sb_2_O_4_/C showed the maximum reversible capacity of 935 mAh/g at the current density of 0.1 A/g, and after 125 cycles, it showed retention of 97.8 % of the initial charge capacity. Thus, it was concluded that Sb_2_O_4_/C material could be used as an anode because it improved the electrochemical properties of NIBs [158].

In October 2019, Saroja *et al.* from IIT Madras worked on the facile synthesis of heteroatom-doped and undoped graphene quantum dots and their application in lithium and Na ion batteries. They prepared graphene quantum dots using a scalable and straightforward approach and chemical vapor deposition method to produce heteroatom-doped graphene quantum dots. They used graphite oxide (by ignoring dialysis bags) at low temperatures to obtain boron-doped and nitrogen-doped graphene quantum dots. Moreover, they analyzed the electrochemical behavior of lithium and sodium ion storage in both doped and undoped graphene quantum dots. They observed that boron-doped GQD (B-GQD) showed a 1097 mAh/g specific capacity for lithium-ion batteries than Na ion batteries, which led to 310 mAh/g at a specific current of 50 mA/g.

Moreover, B-GQD gave 537 Ah/L of volumetric energy density at 0.34 V in lithium-ion batteries and 214 Ah/L volumetric energy density at 0.57 V for Na ion batteries. It also provided fine capacity retention for 500 cycles. According to their work, there were some defects in GQD and doped GQD, which helped improve the electrochemical storage of lithium and sodium ions [159].

Anup Kumar Bera and Seikh M. Yusuf from Bhabha Atomic Research Centre, Mumbai, in January 2020, researched a layered battery material Na_2_Ni_2_TeO_6_. They studied Na ion conduction and its crystal structure as a function of temperature by using impedance spectroscopy and neutron diffraction. The material has an ionic conductivity of σ ≈ 2 × 10^−4^ S/m at 323 K, which changed according to temperature; hence, at 423 K, ionic conductivity became ∼0.03 S/m. Moreover, this conductivity and an average activation energy of about∼0.58 (3) eV for the temperature greater than or equal to 383 K indicated Arrhenius-type behavior. They experimentally studied the site-specific Na-ion conductions using microscopic to study Na ion conduction pathways and verify its molecular dynamics simulation. Two-dimensional Na-ion conduction pathways were confined in a and b planes of Na layers.

Furthermore, its crystal-structural study indicated their higher ionic conductivity value, and the local crystallographic was for site-specific conductivity. The ionic conduction was due to Na ions located at the Na1 and Na2 sites at 500 K. On the other hand, Na ions were at three sites at a temperature above 500 K Na_2_Ni_2_TeO_6_ showed a stable crystal structure in the neutron diffraction at a temperature of 725 K [176].

Sarkar *et al.* from the Indian Institute of Technology Bombay 2020 worked on a material named metal-doped sodium titanium oxide (Na_2_Ti_3_O_7_). They used this material as an anode in NIBs and obtained a higher capacity of about 237 mAh/g at 4000 stable cycles. Moreover, this material had a higher irreversible loss of approximately 53.86%. They wanted to reduce irreversible loss up to 4.11%, so they did chemical shorting for 60 min and obtained the required result. It also improved the Coulombic efficiency up to 36.22%. This method was also used in full-cell (sodium vanadium phosphate vs. hydrogenated metal-doped Na_2_Ti_3_O_7_) and improved its electrochemical properties. Hence, the work was an excellent attempt to reduce the first cycle of irreversible loss of sodium titanate anode [160].

Patel *et al.* from NIT Srinagar, Indian Institute of Science Education and Research Pune, Texas A&M University, College Station, TX, respectively, in the year 2020 worked on Sb_2_Se_3_. They wrapped rGO (reduced graphene oxide) on Sb_2_Se_3_ and analyzed its cyclic instability and rate instability in Na ion conversion. The low weight additive (5 wt.% of rGO) was used, and it yielded a fantastic reversible capacity of 550 mAh/g at a specific current of 100 mA/g. Moreover, they obtained progressive rate performance with 100 % capacity retention at 2 Ag^−1^ current steps. Their work was the first attempt to obtain improved performance and greater mobility in the rGO wrapped composite (Sb_2_Se_3_-rGO) and not in Sb_2_Se_3_. Furthermore, using the GITT method, they got maximum Na ion diffusion coefficients (approx. 30 fold higher) in Sb_2_Se_3_-rGO, which was not obtained in only Sb_2_Se_3_ in the whole operating voltage window. The experiment revealed that the diffusion coefficients of Sb_2_Se_3_ with rGO lie in the 8.0 × 10^−15^ cm^2^ s^−1^ to 2.2 × 10^−12^ cm^2^ s^−1^ range. At the same time, it lies between 1.6 × 10^−15^ cm^2^ s^−1^ to 9.4 × 10^−15^ cm^2^ s^−1^ in the case of only Sb_2_Se_3_ [161].

Mathiyalagan *et al.* from Alagappa University, in 2020, experimented on layered O_3_–Na_0.95_CrO_2_ material and used it as a cathode in Na ion batteries. Using a solid-state reaction, they made this material and studied its thermal, structural, morphological, chemical, and electrochemical properties. The material had a rhombohedral structure with a space group of R3m. Fourier-transform infrared (FTIR) spectroscopy showed the presence of a Na–O bond in the material. Moreover, some quasi-polygonal particles were confirmed using scanning electron microscopy (SEM) and high-resolution transmission electron microscopy (HR-TEM) studies. Using this material in Na ion batteries as a cathode, they got a discharge capacity of 101 mAh/g at 0.1 C at a voltage between 2 to 4 V [145].

Saroja *et al.* from the Indian Institute of Technology Madras, 2019, worked on multi-walled carbon nanotubes by using sulfur nanoparticles inserted into graphene-multiwalled carbon nanotubes (GCNT). It improved the performance of sodium and aluminum ion batteries. They used GCNT/S as an anode in Na ion batteries. As a result, they obtained a specific capacity of approximately 510 mAh/g at a current density of 50 mAg^-1^ with cyclic stability for 2500 cycles which was 42 % more than GCNT. Moreover, aluminum ion batteries also showed 7.2 times more specific capacity than GCNT.

Moreover, when they used GCNT/s as an anode in aluminum ion batteries, they got stable cyclic stability for 600 cycles, 507 mAh/g specific capacity at a current density of 50 mA/g. According to their work, this material was highly durable because tiny sulfur particles provided low volume expansion. Hence, it offered a new approach to improving sodium and aluminum ion batteries' specific capacity and cyclic stability [162].

Kumar et al. from IIT Hyderabad, in 2020, used O_3_-type layered NaNi_0.5_Mn_0.3_Co_0.2_O_2_ cathodes and hard carbon anode derived from dextrose for Na ion coin cell and pouch type cells. They reported that NaNi_0.5_Mn_0.3_Co_0.2_O_2_ cathodes showed ∼136 mAh/g of initial discharge capacity, and after 200 cycles, its capacity retention became 87 mAh/g. In the same way, they got 280 mAh/g reversible capacities at C/10 using hard carbon anodes. They calculated the diffusion coefficient of Na ion as 10^−11^–10^−12^ cm^2^/s, showing good capacity retention and reversibility of the cathode [148].

Thus, from the above discussion, it is evident that different groups are at different heights of research. The need is to tap some industries and exploit the expertise for the overall benefit of the large population.

### 8.3 Recent progress (2021 and beyond)

In 2021, Kali *et al.* from ARCI worked on using waste material for anode application in Na ion batteries. The team used discarded bicycle's rubber tube and transformed that into concentric-shelled disordered carbon (CSC) through a controlled oxidation method. The CSC carbon showed a d spacing of 3.689 nm, and their experimental results displayed a “specific capacity” of around 150 mAh/g at a current rate of 100 mA/g after 100 electrochemical cycles [177]. Pandit *et al.* developed manganese oxide nanorods (α-MnO_2_) for NIBs. The DFT analysis was used to study the sodium intercalation into the α-MnO_2_ matrix. Galvanostatic charge-discharge (GCD) testing was used to determine Na ion insertion/extraction further into the MnO_2_ matrix for potentials of 1–4 V. In the NaPF_6_/EC + DMC (5% FEC) electrolyte, MnO_2_ exhibited significant capacity (109 mAh/g at C/20 current rate) with superior life cyclic stability (58.6 % after 800 cycles) when placed as a cathode for NIBs. MnO_2_'s high crystallinity and hierarchy nanorod structure is accounted for enhanced cycling performance and sustained charge-discharge behaviors [178]. Kumaresan *et al.* from VISTAS, Tamilnadu, in 2021, worked on NIB anode by using biomass-derived hard carbon in a mixed gas environment. The preparation of hard carbon material was done using a gas mixture of nitrogen and carbon dioxide by the thermal activation technique. The discharge/charge capabilities of the prepared hard carbon anode were 413/358 mAh/g. The capacity retaining of the defective hard carbon anode was 86.6 % for the first cycle. For 500 cycles, prepared hard carbon showed better rate performance in Na ion storage [164].

Biswal *et al.* from IIT Kharagpur (2021) prepared the SnO_2_ thin film as an anode by pulsed laser deposition technique to study the NIB performance at different temperatures 3000°C–5000 °C. A high specific capacity of 488 mAh/g was achieved at 3000 °C after 50 cycles. The high specific capacity attained can be attributed to SnO_2_'s sodium (Na) storage mechanisms. Various approaches were used to explore the microstructural variations of thin films, revealing a pure SnO_2_ phase [163]. In 2021, V. Kiran Kumar and their team reported P2-type layered metal oxide Na_0.67_Mn_0.5_Fe_0.5_O_2_ as a cathode material for NIBs. It was synthesized using a basic solution combustion process and thermal process. For 100 cycles, at a 0.1 C rate, the electrochemical efficiency of P2–Na_0.67_Mn_0.5_Fe_0.5_O_2_ cathode indicated a discharge capacity of 166 mAh/g. The modest specific capacity was 111 mAh/g [147].

Arjunan *et al.* from Tamilnadu (2021) used powder from exhausted printer cartridges to make an anode for NIBs. E-waste toner powders were used to create an electrode for a Na-battery. The toner powder was heated to produce a carbon-ferric (C/Fe_3_O_4_) material. At 0.1 C, the composite material based on e-waste had a capacity of 410 mAh/g. The material still produced 280 mAh/g after 100 cycles. The environmental impact of toxic e-waste can be reduced by repurposing it for storing energy [179].

Priyadarshini *et al.* Alagappa University Tamilnadu, in 2021, reported the O_3_-type NaFe_9/20_Cr_9/20_Ti_1/10_O_2_ material and titanium was added as a substitute material that increased the NaO_2_ interlayer spacing of cathode. A primary solid-state reaction was used to make the cathode material. The material showed a 140.63 mAh/g initial discharge capacity, and after 50 cycles, 70.6 % of capacity was retained [180]. *Kalubarme et al.* from Pune in 2021 successfully fabricated the antimony oxide (anode) in the octahedral and rod-like morphologies for NIB. A PVP-assisted hydrothermal method by regulated hydrolysis of antimony precursor was employed. The octahedron-like antimony oxide of the cubic structure produced a high reversible capacity of 623 mAh/g. On the other hand, rod-like antimony oxide showed 65 % capacity, which was retained after 200 cycles. It led to a specific capacity of 202 mAh/g [165]. In 2021, Priyadarshini *et al.* from SRM institute, Tamilnadu, synthesized cathode for NIBs using Na_2_FeP_2_O_7_ material. In the initial cycle, the material with carbon black as a conducting product delivered a discharge potential of 68 mAh/g at 0.1 C. At the initial cycle at the 0.1 C rate, the single-walled carbon nanotube was mixed with material to enhance capacity and achieved a higher discharge capacity of 73 mAh/g [148].

From the above discussion, it is clear that Indian scientists are well-advanced in battery research. Government should make a mission mode project and, with the help of industry, should push the initiative to manufacture Na ion batteries indigenously.

## 9 Conclusion

India's research and development in lithium-ion batteries started much later compared to the other nations of the world. But the establishment setup for making these can be well utilized for Na ion batteries as a different configuration is not required. The research from Indian researchers had shown some good, acceptable results with good specific capacity and high lifetime of electrodes. Carbon-based material can be counted as a target for the future at the industrial level. More focus can be given to using layered materials like selenide to exploit a range of parameters. Also, the solid electrolyte should be also provided more attention as not much work for Na ion in India has been carried out on solid electrolytes.

Recently, internal Faradian data showed that NIBs could be safely charged up to 100% of their capacity within a few minutes. However, it is quite the opposite in the case of LIBs, which use the graphite anode. The graphite cannot be charged rapidly because rapid charge or fast kinetics of Li-ion causes internal heating and can often explode due to lithium plating. Lithium plating occurs when Li-ions get precipitated as lithium, causing an explosion. This way, lithium plating can cause a short circuit internally in the battery.

Furthermore, Na ion batteries may also show sodium plating. With the hard carbon anode and patented electrolyte, Faradian successfully overcame sodium plating and opened the doors to Na ion batteries for fast charging. However, Indian researchers haven't yet focused significantly on sodium plating.

Indian researchers must focus on finding advanced electrode (cathode and anode) materials to achieve higher specific capacities and voltages produced at a practical level with specific energies approaching 200 Wh/kg. Furthermore, more standardized studies should be done on the surface kinetics of similar materials to explore the effect of different electrolytes and binder materials. There are only a few government institutes working in NIB research and development. Private research labs are yet to mark their presence in this research domain. The commercialization of electric vehicles and new industries is needed to lend this field at a greater pace, with state-of-the-art characterization facilities like high field NMR. Having seen the upcoming trends, it can provide a cost-competitive alternative in India soon. India is also lagging in solid-state and thin-film approaches for battery development. Focusing on these will enhance India's overall research and development base for Na ion batteries. To develop new materials for battery application, one needs to deep dive into the science of their chemical structure. Generally, we use diffraction methods to study the crystallinity of the battery materials, and these techniques are best for providing atomic-level images of long-range structures. But, some short-range structures with defects or disorders are difficult to characterize by diffraction techniques. Therefore, solid-state NMR's sensitivity to the local chemical environment turned out to be a useful complementary approach for atomic-level characterization. India, the primary driver of Asia pacific region for expanding the renewable energy market, is anticipated to boost the Na ion revenues soon. In Jan 2022, Reliance New Energy Solar Limited agreed with Faradion to obtain 100 percent equity in shares. Reliance will also use Faradion's sophisticated technology at Dhirubhai Ambani Green Energy Giga Complex at Jamnagar. Other market giants are also expected to enter into this battery research race shortly, and hopefully, NIBs will see the light of the day.

## Declarations

### Author contribution statement

All authors listed have significantly contributed to the development and the writing of this article.

### Funding statement

This work was supported by the Council for Scientific and Industrial Research (CSIR) for the grant of Senior Research Fellowship (S.R.F.). S. Rani was supported by the University Grant Commission (UGC) for JRFship.

### Data availability statement

No data was used for the research described in the article.

### Declaration of interests statement

The authors declare no conflict of interest.

### Additional information

No additional information is available for this paper.
